# *N*′-[4-(Dimethyl­amino)benzyl­idene]acetohydrazide

**DOI:** 10.1107/S1600536809032619

**Published:** 2009-08-22

**Authors:** Wei-Wei Li, Tie-Ming Yu, Wen-Bo Yu, Lu-Ping Lv, Xian-Chao Hu

**Affiliations:** a Department of Chemical Engineering, Hangzhou Vocational and Technical College, Hangzhou 310018, People’s Republic of China; b Research Center of Analysis and Measurement, Zhejiang University of Technology, Hangzhou 310014, People’s Republic of China

## Abstract

The title compound, C_11_H_15_N_3_O, crystallizes with two independent mol­ecules per asymmetric unit which differ slightly in their side-chain orientations: the C=N—N—C torsion angle is −176.2 (3)° in one of the mol­ecules and −179.83 (3)° in the other. Each independent mol­ecule adopts a *trans* configuration with respect to the C=N bond. The two independent mol­ecules are related by a pseudo-inversion center and they exist as a N—H⋯O hydrogen-bonded dimer. The dimers are linked into zigzag chains along [100] by C—H⋯O hydrogen bonds.

## Related literature

For general background to this type of compound, see: Cimerman *et al.* (1997 ▶); Offe *et al.* (1952 ▶); Richardson *et al.* (1988 ▶). For related structures, see: Li & Jian (2008 ▶); Shang *et al.* (2007 ▶); Tamboura *et al.* (2009 ▶).
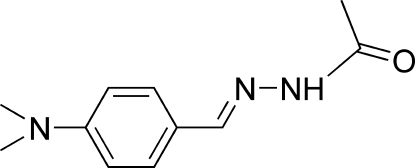


## Experimental

### 

#### Crystal data

C_11_H_15_N_3_O
*M* *_r_* = 205.26
Orthorhombic, 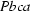

*a* = 8.619 (4) Å
*b* = 20.063 (3) Å
*c* = 26.231 (3) Å
*V* = 4536 (2) Å^3^
*Z* = 16
Mo *K*α radiation
μ = 0.08 mm^−1^
*T* = 223 K
0.25 × 0.21 × 0.19 mm

#### Data collection

Bruker SMART CCD area-detector diffractometer
Absorption correction: multi-scan (*SADABS*; Bruker, 2002 ▶) *T*_min_ = 0.977, *T*_max_ = 0.979
25277 measured reflections
3944 independent reflections
2266 reflections with *I* > 2σ(*I*)
*R*_int_ = 0.086

#### Refinement

*R*[*F* ^2^ > 2σ(*F* ^2^)] = 0.081
*wR*(*F* ^2^) = 0.260
*S* = 1.05
3944 reflections
278 parameters
H-atom parameters constrained
Δρ_max_ = 0.26 e Å^−3^
Δρ_min_ = −0.25 e Å^−3^

### 

Data collection: *SMART* (Bruker, 2002 ▶); cell refinement: *SAINT* (Bruker, 2002 ▶); data reduction: *SAINT*; program(s) used to solve structure: *SHELXS97* (Sheldrick, 2008 ▶); program(s) used to refine structure: *SHELXL97* (Sheldrick, 2008 ▶); molecular graphics: *SHELXTL* (Sheldrick, 2008 ▶); software used to prepare material for publication: *SHELXTL*.

## Supplementary Material

Crystal structure: contains datablocks I, global. DOI: 10.1107/S1600536809032619/ci2860sup1.cif

Structure factors: contains datablocks I. DOI: 10.1107/S1600536809032619/ci2860Isup2.hkl

Additional supplementary materials:  crystallographic information; 3D view; checkCIF report

## Figures and Tables

**Table 1:** Hydrogen-bond geometry (Å, °)

*D*—H⋯*A*	*D*—H	H⋯*A*	*D*⋯*A*	*D*—H⋯*A*
N2—H2⋯O2^i^	0.86	2.09	2.930 (4)	166
N6—H6⋯O1^ii^	0.86	2.04	2.884 (4)	165
C3—H3⋯O1^iii^	0.93	2.56	3.328 (4)	140

Symmetry codes: (i) 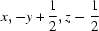; (ii) 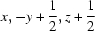; (iii) 
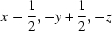
.

**Fig. 1.**
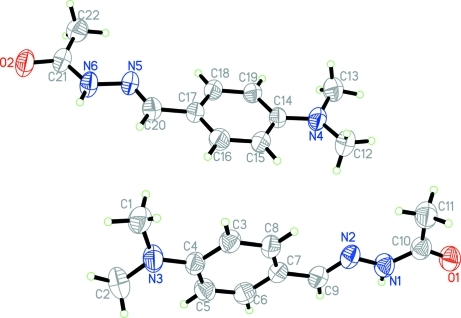
The asymmetric unit of the title compound. Displacement ellipsoids are drawn at the 40% probability level. Dashed lines indicate hydrogen bonds.

**Fig. 2.**
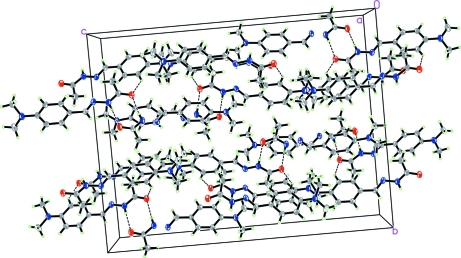
Crystal packing of the title compound. Hydrogen bonds are shown as dashed lines.

## supplementary crystallographic information

### Comment

Schiff bases have attracted much attention due to the possibility of their analytical applications (Cimerman *et al.*,  1997). They are also important ligands, which have been reported to have mild bacteriostatic activity and as potential oral iron-chelating drugs for genetic disorders such as thalassemia (Offe *et al.*, 1952; Richardson *et al.*,  1988). Metal complexes based on Schiff bases have received considerable attention because they can be utilized as model compounds of active centres in various complexes (Tamboura *et al.*,  2009). We report here the crystal structure of the title compound (Fig. 1).

The title compound contains two independent, but almost identical molecules in the asymmetric unit. Each independent molecule adopts a *trans* configuration with respect to the C═N bond. The N1/N2/O1/C9/C10/C11 and N5/N6/O2/C10/C21/C22 planes form dihedral angles of 4.68 (6)° and 6.93 (5)°, respectively, with the C3-C8 and C14-C19 planes. The dihedral angle between the two independent benzene rings is 88.26 (9)°. Bond lengths and angles are comparable to those observed for related structures (Li *et al.*, 2008; Shang *et al.*, 2007).

The two independent molecules are related by a pseudo inversion center, and in the crystal they exist as N—H···O hydrogen-bonded dimers. The dimers are linked into a zigzag chain along the 'a' axis by C—H···O hydrogen bonds. (Table 1).

### Experimental

4-Dimethylaminobenzaldehyde (1.49 g, 0.01 mol) and acetohydrazide (0.74 g, 0.01 mol) were dissolved in stirred methanol (20 ml) and left for 3.5 h at room temperature. The resulting solid was filtered off and recrystallized from ethanol to give the title compound in 87% yield. Single crystals suitable for X-ray analysis were obtained by slow evaporation of an ethanol solution at room temperature (m.p. 490–493 K).

### Refinement

H atoms were positioned geometrically (N-H = 0.86 Å and C-H = 0.93 or 0.96 Å) and refined using a riding model, with *U*_iso_(H) = 1.2*U*_eq_(C,N) and 1.5*U*_eq_(C_methyl_).

### Crystal data

**Table:**

C_11_H_15_N_3_O	*F*(000) = 1760
*M**_r_* = 205.26	*D*_x_ = 1.202 Mg m^−^^3^
Orthorhombic, *P**b**c**a*	Mo *K*α radiation, λ = 0.71073 Å
Hall symbol: -P 2ac 2ab	Cell parameters from 3944 reflections
*a* = 8.619 (4) Å	θ = 1.5–25.0°
*b* = 20.063 (3) Å	µ = 0.08 mm^−^^1^
*c* = 26.231 (3) Å	*T* = 223 K
*V* = 4536 (2) Å^3^	Block, colourless
*Z* = 16	0.25 × 0.21 × 0.19 mm

### Data collection

**Table:**

Bruker SMART CCD area-detector diffractometer	3944 independent reflections
Radiation source: fine-focus sealed tube	2266 reflections with *I* > 2σ(*I*)
graphite	*R*_int_ = 0.086
φ and ω scans	θ_max_ = 25.0°, θ_min_ = 1.6°
Absorption correction: multi-scan (*SADABS*; Bruker, 2002)	*h* = −10→10
*T*_min_ = 0.977, *T*_max_ = 0.979	*k* = −23→23
25277 measured reflections	*l* = −30→27

### Refinement

**Table:**

Refinement on *F*^2^	Secondary atom site location: difference Fourier map
Least-squares matrix: full	Hydrogen site location: inferred from neighbouring sites
*R*[*F*^2^ > 2σ(*F*^2^)] = 0.081	H-atom parameters constrained
*wR*(*F*^2^) = 0.260	*w* = 1/[σ^2^(*F*_o_^2^) + (0.1428*P*)^2^] where *P* = (*F*_o_^2^ + 2*F*_c_^2^)/3
*S* = 1.05	(Δ/σ)_max_ = 0.009
3944 reflections	Δρ_max_ = 0.26 e Å^−^^3^
278 parameters	Δρ_min_ = −0.25 e Å^−^^3^
0 restraints	Extinction correction: *SHELXL97* (Sheldrick, 2008), Fc^*^=kFc[1+0.001xFc^2^λ^3^/sin(2θ)]^-1/4^
Primary atom site location: structure-invariant direct methods	Extinction coefficient: 0.0088 (18)

### Special details

**Table:**

Geometry. All esds (except the esd in the dihedral angle between two l.s. planes) are estimated using the full covariance matrix. The cell esds are taken into account individually in the estimation of esds in distances, angles and torsion angles; correlations between esds in cell parameters are only used when they are defined by crystal symmetry. An approximate (isotropic) treatment of cell esds is used for estimating esds involving l.s. planes.
Refinement. Refinement of F^2^ against ALL reflections. The weighted R-factor wR and goodness of fit S are based on F^2^, conventional R-factors R are based on F, with F set to zero for negative F^2^. The threshold expression of F^2^ > 2sigma(F^2^) is used only for calculating R-factors(gt) etc. and is not relevant to the choice of reflections for refinement. R-factors based on F^2^ are statistically about twice as large as those based on F, and R- factors based on ALL data will be even larger.

### Fractional atomic coordinates and isotropic or equivalent isotropic displacement parameters (Å^2^)

**Table:**

	*x*	*y*	*z*	*U*_iso_*/*U*_eq_	
O1	0.0047 (3)	0.29181 (12)	−0.13459 (9)	0.0780 (8)	
O2	0.1988 (3)	0.06574 (13)	0.42455 (9)	0.0870 (9)	
C1	−0.2222 (5)	0.2853 (2)	0.26167 (14)	0.0876 (12)	
H1A	−0.2352	0.2920	0.2977	0.131*	
H1B	−0.1581	0.2469	0.2560	0.131*	
H1C	−0.3217	0.2784	0.2461	0.131*	
C2	−0.0788 (5)	0.3912 (2)	0.27440 (14)	0.0960 (13)	
H2A	−0.1206	0.3847	0.3080	0.144*	
H2B	−0.1011	0.4357	0.2631	0.144*	
H2C	0.0314	0.3845	0.2752	0.144*	
C3	−0.1822 (4)	0.30188 (16)	0.15390 (12)	0.0642 (9)	
H3	−0.2554	0.2715	0.1657	0.077*	
C4	−0.1112 (4)	0.34523 (16)	0.18857 (12)	0.0609 (9)	
C5	−0.0040 (4)	0.38962 (16)	0.16863 (13)	0.0705 (10)	
H5	0.0450	0.4197	0.1903	0.085*	
C6	0.0317 (4)	0.39019 (17)	0.11734 (13)	0.0711 (10)	
H6A	0.1039	0.4208	0.1053	0.085*	
C7	−0.0365 (4)	0.34675 (16)	0.08350 (12)	0.0602 (9)	
C8	−0.1463 (4)	0.30314 (16)	0.10280 (12)	0.0636 (9)	
H8	−0.1968	0.2741	0.0807	0.076*	
C9	0.0084 (4)	0.34839 (18)	0.03007 (12)	0.0678 (9)	
H9	0.0781	0.3809	0.0194	0.081*	
C10	−0.0398 (4)	0.27958 (17)	−0.09107 (14)	0.0675 (10)	
C11	−0.1489 (5)	0.2237 (2)	−0.08028 (14)	0.0894 (12)	
H11A	−0.1751	0.2236	−0.0447	0.134*	
H11B	−0.1005	0.1821	−0.0890	0.134*	
H11C	−0.2415	0.2292	−0.1002	0.134*	
C12	0.0762 (5)	0.1252 (2)	0.01346 (13)	0.0932 (13)	
H12A	0.1036	0.1176	−0.0215	0.140*	
H12B	0.0797	0.1721	0.0206	0.140*	
H12C	−0.0268	0.1087	0.0196	0.140*	
C13	0.2661 (6)	0.0345 (2)	0.02456 (15)	0.0972 (14)	
H13A	0.2679	0.0387	−0.0119	0.146*	
H13B	0.2141	−0.0060	0.0338	0.146*	
H13C	0.3705	0.0335	0.0373	0.146*	
C14	0.1752 (4)	0.09738 (16)	0.09815 (12)	0.0606 (9)	
C15	0.0720 (5)	0.14273 (16)	0.12098 (12)	0.0684 (10)	
H15	0.0105	0.1699	0.1005	0.082*	
C16	0.0610 (4)	0.14740 (16)	0.17306 (12)	0.0670 (9)	
H16	−0.0087	0.1776	0.1871	0.080*	
C17	0.1510 (4)	0.10824 (15)	0.20548 (11)	0.0579 (9)	
C18	0.2550 (4)	0.06487 (16)	0.18297 (12)	0.0616 (9)	
H18	0.3176	0.0384	0.2036	0.074*	
C19	0.2681 (4)	0.05990 (15)	0.13102 (12)	0.0647 (9)	
H19	0.3408	0.0308	0.1173	0.078*	
C20	0.1307 (4)	0.11238 (16)	0.26024 (12)	0.0642 (9)	
H20	0.0637	0.1445	0.2732	0.077*	
C21	0.2309 (4)	0.05138 (18)	0.37994 (13)	0.0684 (10)	
C22	0.3373 (4)	−0.00500 (18)	0.36685 (13)	0.0772 (11)	
H22A	0.4084	0.0090	0.3408	0.116*	
H22B	0.2775	−0.0421	0.3547	0.116*	
H22C	0.3942	−0.0181	0.3967	0.116*	
N1	−0.0433 (3)	0.30740 (14)	−0.00272 (10)	0.0689 (8)	
N2	0.0109 (3)	0.31648 (14)	−0.05201 (10)	0.0716 (8)	
H2	0.0794	0.3468	−0.0576	0.086*	
N3	−0.1490 (4)	0.34348 (16)	0.23945 (10)	0.0791 (9)	
N4	0.1849 (4)	0.09056 (14)	0.04632 (10)	0.0786 (9)	
N5	0.1997 (3)	0.07432 (14)	0.29129 (10)	0.0647 (8)	
N6	0.1681 (3)	0.08653 (14)	0.34201 (10)	0.0714 (8)	
H6	0.1049	0.1182	0.3495	0.086*	

### Atomic displacement parameters (Å^2^)

**Table:**

	*U*^11^	*U*^22^	*U*^33^	*U*^12^	*U*^13^	*U*^23^
O1	0.0901 (19)	0.0899 (18)	0.0541 (16)	0.0010 (14)	0.0130 (13)	−0.0020 (12)
O2	0.105 (2)	0.107 (2)	0.0488 (15)	0.0166 (16)	0.0105 (14)	0.0072 (12)
C1	0.107 (3)	0.093 (3)	0.063 (2)	0.005 (2)	0.020 (2)	0.0040 (19)
C2	0.098 (3)	0.123 (3)	0.068 (3)	−0.004 (3)	−0.001 (2)	−0.026 (2)
C3	0.066 (2)	0.068 (2)	0.058 (2)	−0.0080 (17)	−0.0004 (17)	0.0067 (16)
C4	0.063 (2)	0.069 (2)	0.050 (2)	0.0069 (17)	−0.0043 (16)	0.0006 (16)
C5	0.079 (3)	0.069 (2)	0.064 (2)	−0.0082 (18)	−0.002 (2)	−0.0084 (17)
C6	0.080 (3)	0.067 (2)	0.067 (2)	−0.0102 (18)	0.003 (2)	0.0036 (17)
C7	0.066 (2)	0.0609 (19)	0.054 (2)	0.0026 (17)	−0.0044 (16)	0.0072 (15)
C8	0.072 (2)	0.068 (2)	0.051 (2)	−0.0029 (18)	−0.0065 (17)	0.0000 (15)
C9	0.078 (3)	0.071 (2)	0.054 (2)	0.0010 (18)	0.0016 (18)	0.0029 (17)
C10	0.069 (2)	0.077 (2)	0.057 (2)	0.0096 (19)	0.0052 (18)	0.0013 (18)
C11	0.098 (3)	0.097 (3)	0.073 (3)	−0.023 (2)	−0.006 (2)	0.006 (2)
C12	0.114 (3)	0.109 (3)	0.057 (2)	0.002 (3)	−0.010 (2)	0.010 (2)
C13	0.135 (4)	0.091 (3)	0.066 (3)	0.011 (3)	0.007 (2)	−0.010 (2)
C14	0.073 (2)	0.0649 (19)	0.0436 (19)	−0.0063 (18)	−0.0001 (17)	−0.0009 (15)
C15	0.088 (3)	0.063 (2)	0.054 (2)	0.0069 (19)	−0.0034 (18)	0.0038 (16)
C16	0.072 (2)	0.068 (2)	0.062 (2)	0.0115 (18)	0.0025 (18)	−0.0035 (16)
C17	0.067 (2)	0.0607 (19)	0.0461 (19)	−0.0017 (16)	0.0002 (16)	0.0010 (14)
C18	0.068 (2)	0.0658 (19)	0.052 (2)	0.0061 (16)	−0.0032 (17)	0.0034 (15)
C19	0.079 (2)	0.0608 (19)	0.054 (2)	0.0037 (17)	0.0038 (18)	−0.0007 (15)
C20	0.067 (2)	0.072 (2)	0.054 (2)	0.0025 (18)	0.0035 (17)	−0.0050 (17)
C21	0.071 (2)	0.087 (2)	0.047 (2)	−0.0010 (19)	0.0036 (18)	0.0094 (18)
C22	0.080 (3)	0.083 (2)	0.068 (2)	0.004 (2)	0.0007 (19)	0.0059 (18)
N1	0.076 (2)	0.0802 (19)	0.0506 (17)	0.0032 (15)	0.0036 (14)	0.0104 (14)
N2	0.082 (2)	0.0794 (18)	0.0530 (18)	−0.0102 (16)	0.0073 (15)	0.0002 (14)
N3	0.095 (2)	0.092 (2)	0.0505 (18)	−0.0090 (18)	0.0027 (16)	−0.0076 (15)
N4	0.105 (3)	0.083 (2)	0.0478 (18)	0.0107 (17)	0.0013 (16)	0.0013 (14)
N5	0.0686 (19)	0.0819 (18)	0.0435 (16)	0.0004 (15)	0.0037 (14)	0.0026 (13)
N6	0.077 (2)	0.091 (2)	0.0465 (17)	0.0135 (16)	0.0070 (15)	0.0007 (14)

### Geometric parameters (Å, °)

**Table:**

O1—C10	1.229 (4)	C12—H12A	0.96
O2—C21	1.236 (4)	C12—H12B	0.96
C1—N3	1.449 (5)	C12—H12C	0.96
C1—H1A	0.96	C13—N4	1.443 (5)
C1—H1B	0.96	C13—H13A	0.96
C1—H1C	0.96	C13—H13B	0.96
C2—N3	1.456 (5)	C13—H13C	0.96
C2—H2A	0.96	C14—N4	1.369 (4)
C2—H2B	0.96	C14—C19	1.396 (5)
C2—H2C	0.96	C14—C15	1.406 (5)
C3—C8	1.376 (4)	C15—C16	1.373 (4)
C3—C4	1.399 (4)	C15—H15	0.93
C3—H3	0.93	C16—C17	1.394 (4)
C4—N3	1.374 (4)	C16—H16	0.93
C4—C5	1.386 (5)	C17—C18	1.381 (4)
C5—C6	1.380 (4)	C17—C20	1.450 (4)
C5—H5	0.93	C18—C19	1.371 (4)
C6—C7	1.376 (5)	C18—H18	0.93
C6—H6A	0.93	C19—H19	0.93
C7—C8	1.384 (5)	C20—N5	1.265 (4)
C7—C9	1.454 (4)	C20—H20	0.93
C8—H8	0.93	C21—N6	1.334 (4)
C9—N1	1.271 (4)	C21—C22	1.496 (5)
C9—H9	0.93	C22—H22A	0.96
C10—N2	1.337 (4)	C22—H22B	0.96
C10—C11	1.491 (5)	C22—H22C	0.96
C11—H11A	0.96	N1—N2	1.387 (3)
C11—H11B	0.96	N2—H2	0.86
C11—H11C	0.96	N5—N6	1.380 (4)
C12—N4	1.450 (5)	N6—H6	0.86
			
N3—C1—H1A	109.5	N4—C13—H13B	109.5
N3—C1—H1B	109.5	H13A—C13—H13B	109.5
H1A—C1—H1B	109.5	N4—C13—H13C	109.4
N3—C1—H1C	109.5	H13A—C13—H13C	109.5
H1A—C1—H1C	109.5	H13B—C13—H13C	109.5
H1B—C1—H1C	109.5	N4—C14—C19	121.6 (3)
N3—C2—H2A	109.5	N4—C14—C15	121.7 (3)
N3—C2—H2B	109.5	C19—C14—C15	116.6 (3)
H2A—C2—H2B	109.5	C16—C15—C14	120.8 (3)
N3—C2—H2C	109.5	C16—C15—H15	119.6
H2A—C2—H2C	109.5	C14—C15—H15	119.6
H2B—C2—H2C	109.5	C15—C16—C17	122.0 (3)
C8—C3—C4	121.5 (3)	C15—C16—H16	119.0
C8—C3—H3	119.2	C17—C16—H16	119.0
C4—C3—H3	119.2	C18—C17—C16	117.1 (3)
N3—C4—C5	122.8 (3)	C18—C17—C20	122.6 (3)
N3—C4—C3	120.7 (3)	C16—C17—C20	120.3 (3)
C5—C4—C3	116.5 (3)	C19—C18—C17	121.6 (3)
C6—C5—C4	121.4 (3)	C19—C18—H18	119.2
C6—C5—H5	119.3	C17—C18—H18	119.2
C4—C5—H5	119.3	C18—C19—C14	121.8 (3)
C7—C6—C5	121.9 (3)	C18—C19—H19	119.1
C7—C6—H6A	119.0	C14—C19—H19	119.1
C5—C6—H6A	119.1	N5—C20—C17	123.1 (3)
C6—C7—C8	117.2 (3)	N5—C20—H20	118.4
C6—C7—C9	119.6 (3)	C17—C20—H20	118.4
C8—C7—C9	123.3 (3)	O2—C21—N6	119.5 (3)
C3—C8—C7	121.4 (3)	O2—C21—C22	122.0 (3)
C3—C8—H8	119.3	N6—C21—C22	118.5 (3)
C7—C8—H8	119.3	C21—C22—H22A	109.5
N1—C9—C7	123.0 (3)	C21—C22—H22B	109.5
N1—C9—H9	118.5	H22A—C22—H22B	109.5
C7—C9—H9	118.5	C21—C22—H22C	109.5
O1—C10—N2	120.0 (3)	H22A—C22—H22C	109.5
O1—C10—C11	121.5 (3)	H22B—C22—H22C	109.5
N2—C10—C11	118.5 (3)	C9—N1—N2	115.3 (3)
C10—C11—H11A	109.5	C10—N2—N1	122.1 (3)
C10—C11—H11B	109.4	C10—N2—H2	119.0
H11A—C11—H11B	109.5	N1—N2—H2	119.0
C10—C11—H11C	109.5	C4—N3—C2	119.7 (3)
H11A—C11—H11C	109.5	C4—N3—C1	121.0 (3)
H11B—C11—H11C	109.5	C2—N3—C1	117.2 (3)
N4—C12—H12A	109.5	C14—N4—C13	120.0 (3)
N4—C12—H12B	109.5	C14—N4—C12	120.2 (3)
H12A—C12—H12B	109.5	C13—N4—C12	116.9 (3)
N4—C12—H12C	109.5	C20—N5—N6	114.9 (3)
H12A—C12—H12C	109.5	C21—N6—N5	123.0 (3)
H12B—C12—H12C	109.5	C21—N6—H6	118.5
N4—C13—H13A	109.5	N5—N6—H6	118.5
			
C8—C3—C4—N3	179.7 (3)	N4—C14—C19—C18	−177.7 (3)
C8—C3—C4—C5	0.5 (5)	C15—C14—C19—C18	2.7 (5)
N3—C4—C5—C6	180.0 (3)	C18—C17—C20—N5	−4.0 (5)
C3—C4—C5—C6	−0.8 (5)	C16—C17—C20—N5	174.0 (3)
C4—C5—C6—C7	−0.3 (5)	C7—C9—N1—N2	179.6 (3)
C5—C6—C7—C8	1.6 (5)	O1—C10—N2—N1	175.8 (3)
C5—C6—C7—C9	−178.4 (3)	C11—C10—N2—N1	−4.8 (5)
C4—C3—C8—C7	0.9 (5)	C9—N1—N2—C10	−176.2 (3)
C6—C7—C8—C3	−1.9 (5)	C5—C4—N3—C2	2.0 (5)
C9—C7—C8—C3	178.1 (3)	C3—C4—N3—C2	−177.1 (3)
C6—C7—C9—N1	176.0 (3)	C5—C4—N3—C1	−160.9 (3)
C8—C7—C9—N1	−4.0 (5)	C3—C4—N3—C1	20.0 (5)
N4—C14—C15—C16	178.1 (3)	C19—C14—N4—C13	13.6 (5)
C19—C14—C15—C16	−2.3 (5)	C15—C14—N4—C13	−166.8 (4)
C14—C15—C16—C17	0.4 (5)	C19—C14—N4—C12	173.7 (3)
C15—C16—C17—C18	1.1 (5)	C15—C14—N4—C12	−6.6 (5)
C15—C16—C17—C20	−177.0 (3)	C17—C20—N5—N6	178.8 (3)
C16—C17—C18—C19	−0.7 (5)	O2—C21—N6—N5	179.0 (3)
C20—C17—C18—C19	177.4 (3)	C22—C21—N6—N5	−1.7 (5)
C17—C18—C19—C14	−1.3 (5)	C20—N5—N6—C21	−179.8 (3)

### Hydrogen-bond geometry (Å, °)

**Table:**

*D*—H···*A*	*D*—H	H···*A*	*D*···*A*	*D*—H···*A*
N2—H2···O2^i^	0.86	2.09	2.930 (4)	166
N6—H6···O1^ii^	0.86	2.04	2.884 (4)	165
C3—H3···O1^iii^	0.93	2.56	3.328 (4)	140
C11—H11A···N1	0.96	2.31	2.791 (5)	110

Symmetry codes: (i) *x*, −*y*+1/2, *z*−1/2; (ii) *x*, −*y*+1/2, *z*+1/2; (iii) *x*−1/2, −*y*+1/2, −*z*.

## References

[bb1] Bruker (2002). *SMART*, *SAINT* and *SADABS* Bruker AXS Inc., Madison, Wisconsin, USA.

[bb2] Cimerman, Z., Galic, N. & Bosner, B. (1997). *Anal. Chim. Acta*, **343**, 145–153.

[bb3] Li, Y.-F. & Jian, F.-F. (2008). *Acta Cryst.* E**64**, o2409. 10.1107/S1600536808037677 PMC2959840 21581378

[bb4] Offe, H. A., Siefen, W. & Domagk, G. (1952). *Z. Naturforsch. Teil B*, **7**, 446–447.

[bb5] Richardson, D., Baker, E., Ponka, P., Wilairat, P., Vitolo, M. L. & Webb, J. (1988). *Thalassemia: Pathophysiology and Management*, Part B, p. 81. New York: Alan R. Liss.

[bb6] Shang, Z.-H., Zhang, H.-L. & Ding, Y. (2007). *Acta Cryst.* E**63**, o3394.

[bb7] Sheldrick, G. M. (2008). *Acta Cryst.* A**64**, 112–122. 10.1107/S0108767307043930 18156677

[bb8] Tamboura, F. B., Gaye, M., Sall, A. S., Barry, A. H. & Bah, Y. (2009). *Acta Cryst.* E**65**, m160–m161. 10.1107/S1600536809000105 PMC2968131 21581771

